# Prognostic implications of the extent of downstaging after neoadjuvant therapy for oesophageal adenocarcinoma and oesophageal squamous cell carcinoma

**DOI:** 10.1093/bjsopen/zrad042

**Published:** 2023-06-21

**Authors:** Sivesh K Kamarajah, Sheraz R Markar, Donald Low, Alexander W Phillips

**Affiliations:** Department of Upper Gastrointestinal Surgery, Queen Elizabeth Hospital Birmingham, University Hospitals Birmingham NHS Trust, Birmingham, UK; Academic Department of Surgery, Institute of Applied Health Research, College of Medical and Dental Sciences, University of Birmingham, Birmingham, UK; Department of Surgery & Cancer, Imperial College London, London, UK; Department of Molecular Medicine & Surgery, Karolinska Institutet, Stockholm, Sweden; Nuffield Department of Surgery, University of Oxford, Oxford, UK; Department of Thoracic Surgery, Virginia Mason Medical Center, Seattle, Washington, USA; Northern Oesophagogastric Unit, Royal Victoria Infirmary, Newcastle University Trust Hospitals, Newcastle upon Tyne, UK; School of Medical Education, Newcastle University, Newcastle upon Tyne, UK

## Abstract

**Background:**

There are few data evaluating the extent of downstaging in patients with oesophageal adenocarcinoma and oesophageal squamous cell carcinoma and the difference in outcomes for a similar pathological stage in neoadjuvant-naive patients. The aim of this study was to characterize the prognostic value of downstaging extent in patients receiving neoadjuvant therapy for oesophageal cancer.

**Methods:**

Oesophageal adenocarcinoma and oesophageal squamous cell carcinoma patients receiving either neoadjuvant chemotherapy or neoadjuvant chemoradiotherapy between 2004 and 2017 were identified from the National Cancer Database. The extent of downstaging was defined as the extent of migration between groups (for example stage IVa to IIIb = one stage). Cox multivariable regression was used to produce adjusted models for downstaging extent.

**Results:**

Of 13 594 patients, 11 355 with oesophageal adenocarcinoma and 2239 with oesophageal squamous cell carcinoma were included. In oesophageal adenocarcinoma, patients with downstaged disease by three or more stages (hazards ratio (HR) 0.40, 95 per cent c.i. 0.36 to 0.44, *P* < 0.001), two stages (HR 0.43, 95 per cent c.i. 0.39 to 0.48, *P* < 0.001), or one stage (HR 0.57, 95 per cent c.i. 0.52 to 0.62, *P* < 0.001) had significantly longer survival than those with upstaged disease in adjusted analyses. In oesophageal squamous cell carcinoma, patients with downstaged disease by three or more stages had significantly longer survival than those with less downstaged disease, no change, or upstaged disease. Patients with downstaged disease by three or more stages (HR 0.55, 95 per cent c.i. 0.43 to 0.71, *P* < 0.001), two stages (HR 0.58, 95 per cent c.i. 0.46 to 0.73, *P* < 0.001), or one stage (HR 0.69, 95 per cent c.i. 0.55 to 0.86, *P* = 0.001) had significantly longer survival than those with upstaged disease in adjusted analyses.

**Conclusion:**

The extent of downstaging is an important prognosticator, whereas the optimal neoadjuvant therapy remains controversial. Identifying biomarkers associated with response to neoadjuvant regimens may permit individualized treatment.

## Teaser

Extent of downstaging is an important prognosticator. The optimal neoadjuvant therapy remains controversial. Identifying biomarkers associated with response to neoadjuvant regimens may permit individualized treatment.

## Introduction

Multimodality treatment with neoadjuvant therapy (NAT) followed by resection remains the best curative option for patients with locally advanced oesophageal cancer^1,2^. Currently, clinicians are guided in their decision-making and the treatment offered by the clinical stage of disease. However, increasing evidence has suggested that pathological stage is a better guide to prognosis than the information obtained by pretreatment staging modalities. This has also been shown to be true in patients receiving NAT (that is neoadjuvant chemotherapy (nCT) or neoadjuvant chemoradiotherapy (nCRT))^1,3^.

nCRT has been shown to have a relatively high rate of pathological complete regression in patients and, equally, the FLOT4 study showed pathological complete regression in 17.0 per cent of patients, although this included gastric adenocarcinoma^1,4^. It remains unclear how important this factor is in the final prognosis and indeed the impact of degree of downstaging on survival. More recently there has been some suggestion that tumour regression in lymph nodes is an important prognosticator^5^. Whilst clinical staging is necessary for initial treatment planning, pathological stage and the impact of any NAT could potentially influence the use of adjuvant modalities. It may also impact on patient decision-making through improved prognostication.

The aim of this study was to determine the impact of downstaging on overall survival in a large, unselected cohort of patients. In addition, stratified analyses by tumour histology (that is oesophageal adenocarcinoma (OAC) and oesophageal squamous cell carcinoma (OSCC)) and type of NAT (that is nCT and nCRT) were performed to evaluate extent of downstaging in these subgroups on long-term survival.

## Methods

### Data source

The National Cancer Database (NCDB) is a joint project of the Commission on Cancer (CoC) of the American College of Surgeons and the American Cancer Society^6,7^. Data from over 1500 CoC-accredited hospitals are gathered by clinicians (that is surgeons and oncologists) involved in patient care, which captures up to more than 70 per cent^8^ of all newly diagnosed cancers across the USA. Details on demographics, facility type and location, clinicopathological characteristics, treatment, and outcomes are available.

### Study population

NCDB was used to identify all patients older than 18 years diagnosed with non-metastatic OAC and OSCC undergoing oesophagectomy with curative intent between 2004 and 2016. The International Classification of Disease for Oncology, Third Edition (ICD-O-3) was used to select adenocarcinoma and to exclude other histologies (ICD-O-3 morphology codes 8240–8248). Patients with concomitant cancer diagnoses and those with missing data on receipt of perioperative chemotherapy were excluded.

The following patient-level characteristics were analysed: age (36–50, 51–65, 66–80, older than 80 years), race (white, black, other), Charlson co-morbidity index (CCI), year of diagnosis, insurance status (Medicaid/Medicare, private insurance, uninsured), zip code-level education status (less than 7.0, 7.0–12.9, 13.0–20.9, greater than or equal to 21.0 per cent (see below)), nodal status (N0, N1, N2), tumour grade/differentiation (well, moderate, poor, anaplastic), margin status (negative, positive), and lymphovascular invasion (absent, present). Education level was determined by matching each patient’s zip code at the time of diagnosis with data derived from the 2012 American Community Survey on the percentage of people aged 25 years and older who had not graduated high school (earned a high school diploma). Education categories were based on equally proportioned quartiles, defined as: 21 per cent or more had not graduated high school (lowest education level); 13–20.9 per cent had not graduated high school; 7–12.9 per cent had not graduated high school; and less than 7 per cent had not graduated high school (highest education level). The Eighth Edition of the American Joint Commission on Cancer (AJCC) staging system was used for both T and N classifications. Margin status was defined according to the guidelines of the College of American Pathologists^9^.

### Definition of downstaging

Patients were regarded as having been downstaged if the stage derived from analysis of the pathology specimen was earlier than the clinical stage. Stage movement was regarded as having occurred between any group (for example stage IVa to IIIb = one stage, and stage IVa to IIIa = two stages), as previously described^3^.

### Comparison with straight-to-surgery patients

In addition to investigating the impact of downstaging, a comparison on outcomes was carried out among patients who underwent surgery, but did not receive nCRT. This included a cohort of patients who were pT2 N0, as NAT is not routinely offered to these patients. Similar comparisons were made among patients who did not receive NAT and had a more advanced pathological stage (pT3 N0 *versus* ypT3 N0; pT3 N1 *versus* ypT3 N1; pT3/4 N2/3 *versus* ypT3/4 N2/3).

### Statistical analysis

Categorical variables were compared using the chi-squared test. Non-normally distributed data were analysed using the Mann–Whitney *U* test. Comparisons were made for the main explanatory variable, namely the extent of downstaging (that is upstaged, no change, or downstaged by one stage, two stages, or three or more stages). Survival was estimated using Kaplan–Meier survival curves and compared using the log rank test. Multivariable analyses used Cox proportional hazards models to adjust for clinically relevant variables to produce adjusted HR and 95 per cent confidence intervals. *P* < 0.050 was considered to be statistically significant. Data analysis was performed using R Foundation Statistical Software (R 3.2.2) with the TableOne, ggplot2, Hmisc, Matchit, and survival packages (R Foundation for Statistical Computing, Vienna, Austria) as previously described^10,11^. This study was exempt from Institutional Review Board approval.

## Results

### Oesophageal adenocarcinoma

#### Patient characteristics

This study included 11 355 patients with OAC who underwent oesophagectomy after NAT, of which 18 per cent (1982 patients) were downstaged by three or more stages, followed by 19 per cent (2133 patients) who were downstaged by two stages, and 30 per cent (3383 patients) who were downstaged by one stage. The baseline characteristics of patients by extent of downstaging are presented in *Table 1*. Rates of downstaging by three or more stages were significantly higher in the more recent time intervals and in patients receiving nCRT. Further, there were significantly higher rates of margin-negative resections (three or more stages *versus* two stages *versus* one stage *versus* upstaged *versus* no change—99 *versus* 98 *versus* 95 *versus* 91 *versus* 84 per cent respectively, *P* < 0.001) and lower rates of lymphovascular invasion (three or more stages *versus* two stages *versus* one stage *versus* no change *versus* upstaged—1 *versus* 4 *versus* 10 *versus* 20 *versus* 25 per cent respectively, *P* < 0.001) with greater levels of downstaging.

**Table 1:** Baseline hospital-, patient-, and pathology-level characteristics in patients undergoing neoadjuvant therapy and oesophagectomy for oesophageal adenocarcinoma by extent of downstaging

		Upstaged	No change	Downstaged by one stage	Downstaged by two stages	Downstaged by three or more stages	*P*
Facility type	Community	296 (38.7)	1052 (34.0)	1041 (30.8)	653 (30.6)	631 (31.8)	<0.001
	Integrated	111 (14.5)	442 (14.3)	462 (13.7)	311 (14.6)	270 (13.6)	
	Academic	357 (46.7)	1599 (51.7)	1880 (55.6)	1169 (54.8)	1081 (54.5)	
Facility location	North-east	169 (22.1)	780 (25.2)	858 (25.4)	550 (25.8)	476 (24.0)	0.091
	Midwest	242 (31.7)	943 (30.5)	1057 (31.2)	679 (31.8)	684 (34.5)	
	South	242 (31.7)	912 (29.5)	996 (29.4)	638 (29.9)	561 (28.3)	
	West	111 (14.5)	458 (14.8)	472 (14.0)	266 (12.5)	261 (13.2)	
Hospital distance (miles)	<12.5	345 (45.2)	1357 (43.9)	1457 (43.1)	939 (44.0)	880 (44.4)	0.958
	12.5–49.9	252 (33.0)	1065 (34.4)	1155 (34.1)	730 (34.2)	673 (34.0)	
	≥50	167 (21.9)	671 (21.7)	771 (22.8)	464 (21.8)	429 (21.6)	
Year of diagnosis	2004–2005	50 (6.5)	210 (6.8)	180 (5.3)	94 (4.4)	50 (2.5)	<0.001
	2006–2007	55 (7.2)	255 (8.2)	230 (6.8)	111 (5.2)	89 (4.5)	
	2008–2009	94 (12.3)	368 (11.9)	303 (9.0)	167 (7.8)	134 (6.8)	
	2010–2011	138 (18.1)	532 (17.2)	613 (18.1)	337 (15.8)	329 (16.6)	
	2012–2013	169 (22.1)	661 (21.4)	714 (21.1)	494 (23.2)	461 (23.3)	
	2014–2015	90 (11.8)	355 (11.5)	443 (13.1)	293 (13.7)	288 (14.5)	
	2016–2017	168 (22.0)	712 (23.0)	900 (26.6)	637 (29.9)	631 (31.8)	
Age at diagnosis (years)	18–35	11 (1.4)	27 (0.9)	20 (0.6)	12 (0.6)	16 (0.8)	0.024
	36–50	88 (11.6)	308 (10.0)	304 (9.0)	200 (9.4)	188 (9.5)	
	51–65	386 (50.7)	1600 (51.8)	1719 (50.9)	1043 (48.9)	980 (49.5)	
	66–80	267 (35.1)	1110 (35.9)	1290 (38.2)	859 (40.3)	765 (38.6)	
	>80	9 (1.2)	43 (1.4)	45 (1.3)	17 (0.8)	31 (1.6)	
Sex	Male	687 (89.9)	2739 (88.6)	3010 (89.0)	1870 (87.7)	1738 (87.7)	0.292
	Female	77 (10.1)	354 (11.4)	373 (11.0)	263 (12.3)	244 (12.3)	
Race	White	738 (96.6)	2961 (95.7)	3270 (96.7)	2075 (97.3)	1919 (96.8)	0.037
	Other	26 (3.4)	132 (4.3)	113 (3.3)	58 (2.7)	63 (3.2)	
CCI	0	542 (70.9)	2243 (72.5)	2443 (72.2)	1524 (71.4)	1411 (71.2)	0.669
	1–2	210 (27.5)	798 (25.8)	879 (26.0)	562 (26.3)	542 (27.3)	
	>2	12 (1.6)	52 (1.7)	61 (1.8)	47 (2.2)	29 (1.5)	
Insurance status	Medicare	296 (39.2)	1244 (40.9)	1403 (42.2)	924 (44.2)	839 (43.0)	0.138
	Medicaid	35 (4.6)	141 (4.6)	171 (5.1)	88 (4.2)	81 (4.1)	
	Private insurance	401 (53.0)	1545 (50.8)	1627 (48.9)	1014 (48.5)	978 (50.1)	
	Not insured/other	24 (3.2)	110 (3.6)	127 (3.8)	65 (3.1)	54 (2.8)	
Education level (%)	≥21	104 (13.6)	575 (18.6)	632 (18.7)	464 (21.8)	419 (21.1)	<0.001
	13–20.9	210 (27.5)	717 (23.2)	766 (22.6)	477 (22.4)	391 (19.7)	
	7–12.9	260 (34.0)	1054 (34.1)	1133 (33.5)	697 (32.7)	683 (34.5)	
	<7	190 (24.9)	747 (24.2)	852 (25.2)	495 (23.2)	489 (24.7)	
Medical income (€)	≤€47 999	258 (33.8)	1061 (34.3)	1123 (33.2)	728 (34.1)	667 (33.7)	0.002
	€48 000−62 999	239 (31.3)	854 (27.6)	885 (26.2)	511 (24.0)	507 (25.6)	
	≥€63 000	267 (34.9)	1178 (38.1)	1375 (40.6)	894 (41.9)	808 (40.8)	
Residence	Metro	591 (77.4)	2347 (75.9)	2579 (76.2)	1638 (76.8)	1520 (76.7)	0.044
	Urban	141 (18.5)	557 (18.0)	559 (16.5)	342 (16.0)	330 (16.6)	
	Rural	32 (4.2)	189 (6.1)	245 (7.2)	153 (7.2)	132 (6.7)	
Neoadjuvant therapy	nCT	76 (9.9)	245 (7.9)	217 (6.4)	122 (5.7)	70 (3.5)	<0.001
	nCRT	688 (90.1)	2848 (92.1)	3166 (93.6)	2011 (94.3)	1912 (96.5)	
Tumour grade	Well	18 (2.4)	113 (3.7)	116 (3.4)	141 (6.6)	74 (3.7)	<0.001
	Moderate	275 (36.0)	1070 (34.6)	1117 (33.0)	1060 (49.7)	710 (35.8)	
	Poor	361 (47.3)	1497 (48.4)	1627 (48.1)	658 (30.8)	799 (40.3)	
	Anaplastic	110 (14.4)	413 (13.4)	523 (15.5)	274 (12.8)	399 (20.1)	
AJCC clinical overall stage	I	144 (18.8)	227 (7.3)	76 (2.2)	0 (0.0)	0 (0.0)	<0.001
	IIA	40 (5.2)	61 (2.0)	67 (2.0)	50 (2.3)	0 (0.0)	
	IIB	187 (24.5)	288 (9.3)	384 (11.4)	259 (12.1)	0 (0.0)	
	III	393 (51.4)	2348 (75.9)	2419 (71.5)	1485 (69.6)	1497 (75.5)	
	IVA	0 (0.0)	169 (5.5)	437 (12.9)	339 (15.9)	485 (24.5)	
Regional nodes examined (*n*)	<15	389 (50.9)	1676 (54.2)	1881 (55.6)	1223 (57.3)	1182 (59.6)	<0.001
	≥15	375 (49.1)	1417 (45.8)	1502 (44.4)	910 (42.7)	800 (40.4)	
Margin status	Positive	126 (16.5)	271 (8.8)	165 (4.9)	49 (2.3)	13 (0.7)	<0.001
	Negative	638 (83.5)	2822 (91.2)	3218 (95.1)	2084 (97.7)	1969 (99.3)	
Lymphovascular invasion	Absent	233 (30.5)	1053 (34.0)	1590 (47.0)	1176 (55.1)	829 (41.8)	<0.001
	Present	191 (25.0)	631 (20.4)	350 (10.3)	93 (4.4)	23 (1.2)	
	Unknown	340 (44.5)	1409 (45.6)	1443 (42.7)	864 (40.5)	1130 (57.0)	
Length of stay, days	Median (i.q.r.)	9.0 (7.0–10.0)	9.0 (8.0–11.0)	9.0 (7.0–10.0)	9.0 (7.0–11.0)	9.0 (7.0–11.0)	0.119

Values are *n* (%) unless otherwise indicated. CCI, Charlson co-morbidity index; nCT, neoadjuvant chemotherapy; nCRT, neoadjuvant chemoradiotherapy; AJCC, American Joint Commission on Cancer; i.q.r., interquartile range.

#### Overall survival

**Fig. 1:**
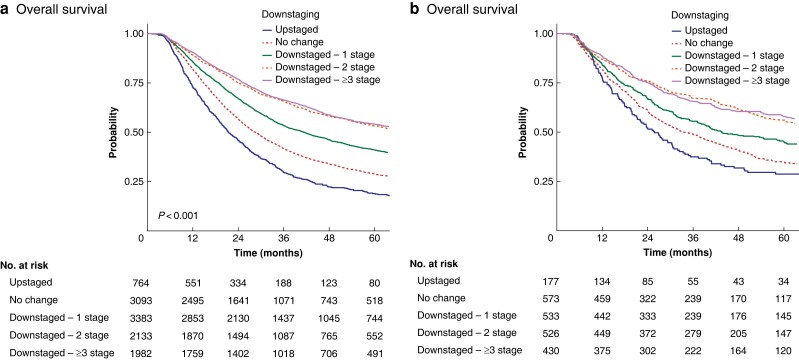
Overall survival of patients with oesophageal cancer receiving neoadjuvant therapy and oesophagectomy **a** Oesophageal adenocarcinoma. **b** Oesophageal squamous cell carcinoma.

Patients who had downstaged disease by three or more stages had significantly longer survival than those with downstaged disease by two stages or one stage and no change or upstaged disease (median of 77.0 *versus* 67.6 *versus* 41.3 *versus* 28.4 *versus* 21.4 months respectively, *P* < 0.001) (*Fig. 1a*). In adjusted analysis, patients with downstaged disease by three or more stages (HR 0.40, 95 per cent c.i. 0.36 to 0.44, *P* < 0.001), two stages (HR 0.43, 95 per cent c.i. 0.39 to 0.48, *P* < 0.001), or one stage (HR 0.57, 95 per cent c.i. 0.52 to 0.62, *P* < 0.001) had significantly longer survival than those with upstaged disease (*Table 2*). Other independent adverse prognostic factors were receipt of nCT, the presence of poor tumour grade, 15 or more lymph nodes examined, margin-negative resections, and absent lymphovascular invasion (*Table S1*). Sensitivity analyses were performed for both nCRT and nCT, which demonstrated consistent results (*Table S2*). Sensitivity analyses were performed by receipt of adjuvant therapy, which demonstrated consistent results (*Table S3*).

**Table 2:** Adjusted multivariable Cox regression on overall survival in patients undergoing neoadjuvant therapy and oesophagectomy for oesophageal adenocarcinoma and oesophageal squamous cell carcinoma by degree of downstaging

	Patients	Median (95% c.i.) overall survival (months)	HR (95% c.i.)	*P*
**Oesophageal adenocarcinoma**				
Upstaged	764 (6.7)	21.4 (19.9,23.8)	Reference	
No change	3093 (27.2)	28.4 (27.0,29.9)	0.76 (0.70,0.84)	<0.001
Downstaged by one stage	3383 (29.8)	41.3 (38.2,43.9)	0.57 (0.52,0.62)	<0.001
Downstaged by two stages	2133 (18.8)	67.6 (62.6,75.4)	0.43 (0.39,0.48)	<0.001
Downstaged by three or more stages	1982 (17.5)	77.0 (65.0,89.5)	0.40 (0.36,0.44)	<0.001
**Oesophageal squamous cell carcinoma**				
Upstaged	177 (7.9)	25.8 (21.3,30.9)	Reference	
No change	573 (25.6)	33.3 (29.1,40.6)	0.86 (0.69,1.06)	0.100
Downstaged by one stage	533 (23.8)	43.6 (38.2,60.0)	0.69 (0.55,0.86)	0.001
Downstaged by two stages	526 (23.5)	72.6 (62.6,97.4)	0.58 (0.46,0.73)	<0.001
Downstaged by three or more stages	430 (19.2)	78.8 (69.4,114.1)	0.55 (0.43,0.71)	<0.001

Values are *n* (%) unless otherwise indicated.

#### Sensitivity analysis by downstaging of T3/4 N+

**Fig. 2:**
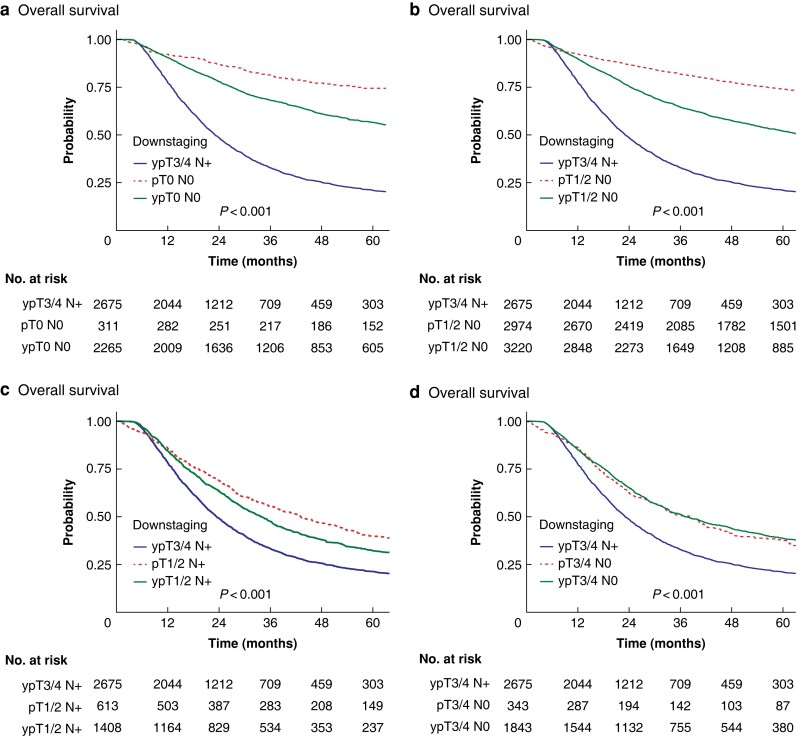
Comparison of patients with oesophageal adenocarcinoma receiving neoadjuvant therapy and oesophagectomy, downstaged from cT3/4 N+ **a** ypT0 N0/pT0 N0. **b** ypT1/2 N0/pT1/2 N0. **c** ypT1/2 N+/pT1/2 N+. **d** ypT3/4 N0/pT3/4 N0. Control groups are represented by cT3/4 N+ to ypT3/4 N+ (not downstaged) and stage-matched controls.

Sensitivity analyses of patients initially clinically staged as T3/4 N+ (cT3/4 N+) who received NAT and were downstaged to ypT0 N0 (*Fig. 2a*), ypT1/2 N0 (*Fig. 2b*), ypT1/2 N+ (*Fig. 2c*), or ypT3/4 N0 (*Fig. 2d*) were performed. In each survival graph, the two control curves represent stage-matched patients who were not administered nCT (pTNM) and patients who were not downstaged by chemotherapy (that is non-responders who were still ypT3/4 N+ after surgical resection). In all of these survival analyses, a significant survival benefit was seen in NAT responders *versus* non-responders, whereas no difference was observed between responders and stage-matched neoadjuvant-naive controls (*Table 3*).

**Table 3:** Overall survival in patients undergoing neoadjuvant therapy and oesophagectomy for oesophageal adenocarcinoma and oesophageal squamous cell carcinoma downstaged from cT3/4 N+ disease with comparison of patients undergoing oesophagectomy only

	Oesophageal adenocarcinoma		Oesophageal squamous cell carcinoma	
	Patients	Median (95% c.i.) overall survival (months)	Patients	Median (95% c.i.) overall survival (months)
**Control**				
ypT3/4 N+	2675	23.1 (22.2,24.3)	216	20.6 (18.3,26.8)
**Group 1**				
pT0 N0	311	141.8 (125.8,NR)	71	133.3 (79.6,NR)
ypT0 N0	2265	83.7 (73.0,94.7)	494	81.4 (69.7,120.1)
**Group 2**				
pT1/2 N0	2974	153.6 (139.2,NR)	356	96.7 (82.9,124.5)
ypT1/2 N0	3220	65.4 (60.2,69.2)	286	73.0 (53.6,112.7)
**Group 3**				
pT1/2 N+	613	42.6 (37.4,50.5)	56	88.1 (43.3,NR)
ypT1/2 N+ (downstaged)	521	36.0 (30.9,39.8)	32	25.1 (17.0,40.3)
ypT1/2 N+ (no downstaging)	887	32.7 (29.5,35.0)	84	25.8 (21.6,33.2)
**Group 4**				
pT3/4 N0	343	38.6 (31.9,44.1)	71	18.7 (13.9,53.4)
ypT3/4 N0 (downstaged)	1627	38.3 (35.2,42.7)	202	35.1 (26.7,57.9)
ypT3/4 N0 (no downstaging)	216	31.4 (26.1,39.9)	17	18.4 (11.6,NR)

Values are *n* unless otherwise indicated. NR, not reached.

#### Sensitivity analysis by margin-negative resections

Sensitivity analyses in patients with margin-negative resections (10 731 patients) demonstrated patients who had downstaged disease by three or more stages had significantly longer survival than those with downstaged disease by two stages or one stage and no change or upstaged disease (median of 77.0 *versus* 67.9 *versus* 42.5 *versus* 29.6 *versus* 23.4 months respectively, *P* < 0.001), consistent on adjusted analysis (*Table S4*). Similar results were seen in patients receiving nCRT and nCT (*Table S4*).

### Oesophageal squamous cell carcinoma

#### Patient characteristics

This study included 2239 patients with OSCC who underwent oesophagectomy after NAT, of which 19 per cent (430 patients) were downstaged by three or more stages, followed by 24 per cent (526 patients) who were downstaged by two stages and 24 per cent (533 patients) who were downstaged by one stage. The baseline characteristics of patients by extent of downstaging are presented in *Table 4*. Rates of downstaging of three or more stages were significantly higher in patients with poor/anaplastic disease and in patients receiving nCRT. Further, patients with downstaging of three or more stages had significantly higher rates of margin-negative resections (99 *versus* 98 *versus* 94 *versus* 91 *versus* 86 per cent respectively, *P* < 0.001) and presence of lymphovascular invasion (0 *versus* 2 *versus* 5 *versus* 11 *versus* 16 per cent respectively, *P* < 0.001) compared with patients with downstaging by two stages or one stage and no change or upstaged disease.

**Table 4:** Baseline clinicopathological characteristics in patients undergoing neoadjuvant therapy and oesophagectomy for oesophageal squamous cell carcinoma by extent of downstaging

		Upstaged	No change	Downstaged by one stage	Downstaged by two stages	Downstaged by three or more stages	*P*
Facility type	Community	69 (39.0)	167 (29.1)	165 (31.0)	159 (30.2)	121 (28.1)	0.200
	Integrated	18 (10.2)	71 (12.4)	83 (15.6)	70 (13.3)	55 (12.8)	
	Academic	90 (50.8)	335 (58.5)	285 (53.5)	297 (56.5)	254 (59.1)	
Facility location	North-east	48 (27.1)	148 (25.8)	145 (27.2)	119 (22.6)	115 (26.7)	0.100
	Midwest	53 (29.9)	143 (25.0)	142 (26.6)	145 (27.6)	135 (31.4)	
	South	51 (28.8)	187 (32.6)	181 (34.0)	185 (35.2)	139 (32.3)	
	West	25 (14.1)	95 (16.6)	65 (12.2)	77 (14.6)	41 (9.5)	
Hospital distance (miles)	<12.5	104 (58.8)	294 (51.3)	272 (51.0)	279 (53.0)	205 (47.7)	0.100
	12.5–49.9	51 (28.8)	163 (28.4)	162 (30.4)	142 (27.0)	149 (34.7)	
	≥50	22 (12.4)	116 (20.2)	99 (18.6)	105 (20.0)	76 (17.7)	
Year of diagnosis	2004–2005	20 (11.3)	47 (8.2)	42 (7.9)	27 (5.1)	22 (5.1)	<0.001
	2006–2007	10 (5.6)	54 (9.4)	49 (9.2)	28 (5.3)	16 (3.7)	
	2008–2009	26 (14.7)	50 (8.7)	40 (7.5)	42 (8.0)	34 (7.9)	
	2010–2011	26 (14.7)	120 (20.9)	119 (22.3)	83 (15.8)	77 (17.9)	
	2012–2013	35 (19.8)	127 (22.2)	107 (20.1)	132 (25.1)	110 (25.6)	
	2014–2015	26 (14.7)	60 (10.5)	52 (9.8)	74 (14.1)	51 (11.9)	
	2016–2017	34 (19.2)	115 (20.1)	124 (23.3)	140 (26.6)	120 (27.9)	
Age at diagnosis (years)	18–35	1 (0.6)	5 (0.9)	7 (1.3)	3 (0.6)	0 (0.0)	0.100
	36–50	12 (6.8)	55 (9.6)	46 (8.7)	44 (8.4)	45 (10.5)	
	51–65	108 (61.0)	272 (47.5)	282 (53.1)	277 (52.7)	235 (54.7)	
	66–80	52 (29.4)	234 (40.8)	192 (36.2)	199 (37.8)	144 (33.5)	
	>80	4 (2.3)	7 (1.2)	4 (0.8)	3 (0.6)	6 (1.4)	
Sex	Male	113 (63.8)	348 (60.7)	335 (62.9)	296 (56.3)	263 (61.2)	0.200
	Female	64 (36.2)	225 (39.3)	198 (37.1)	230 (43.7)	167 (38.8)	
Race	White	126 (71.2)	445 (77.7)	419 (78.6)	396 (75.3)	339 (78.8)	0.200
	Other	51 (28.8)	128 (22.3)	114 (21.4)	130 (24.7)	91 (21.2)	
CCI	0	142 (80.2)	457 (79.8)	414 (77.7)	403 (76.6)	326 (75.8)	0.800
	1–2	33 (18.6)	110 (19.2)	114 (21.4)	114 (21.7)	96 (22.3)	
	>2	2 (1.1)	6 (1.0)	5 (0.9)	9 (1.7)	8 (1.9)	
Insurance status	Medicare	66 (38.6)	267 (47.1)	208 (39.6)	215 (42.1)	161 (38.1)	0.100
	Medicaid	18 (10.5)	47 (8.3)	50 (9.5)	63 (12.3)	40 (9.5)	
	Private insurance	77 (45.0)	233 (41.1)	241 (45.9)	216 (42.3)	207 (48.9)	
	Not insured/other	10 (5.8)	20 (3.5)	26 (5.0)	17 (3.3)	15 (3.5)	
Education level (%)	≥21	50 (28.2)	136 (23.7)	123 (23.1)	149 (28.3)	112 (26.0)	0.100
	13–20.9	35 (19.8)	124 (21.6)	141 (26.5)	105 (20.0)	82 (19.1)	
	7–12.9	48 (27.1)	168 (29.3)	161 (30.2)	168 (31.9)	143 (33.3)	
	<7	44 (24.9)	145 (25.3)	108 (20.3)	104 (19.8)	93 (21.6)	
Medical income (€)	≤€47 999	65 (36.7)	209 (36.5)	221 (41.5)	209 (39.7)	141 (32.8)	0.300
	€48 000–62 999	45 (25.4)	139 (24.3)	127 (23.8)	116 (22.1)	111 (25.8)	
	≥€63 000	67 (37.9)	225 (39.3)	185 (34.7)	201 (38.2)	178 (41.4)	
Residence	Metro	146 (82.5)	470 (82.0)	421 (79.0)	414 (78.7)	349 (81.2)	0.800
	Urban	22 (12.4)	68 (11.9)	77 (14.4)	81 (15.4)	55 (12.8)	
	Rural	9 (5.1)	35 (6.1)	35 (6.6)	31 (5.9)	26 (6.0)	
Neoadjuvant therapy	nCT	17 (9.6)	36 (6.3)	35 (6.6)	16 (3.0)	9 (2.1)	<0.001
	nCRT	160 (90.4)	537 (93.7)	498 (93.4)	510 (97.0)	421 (97.9)	
Tumour grade	Well	13 (7.3)	27 (4.7)	33 (6.2)	42 (8.0)	38 (8.8)	<0.001
	Moderate	85 (48.0)	240 (41.9)	236 (44.3)	252 (47.9)	168 (39.1)	
	Poor	54 (30.5)	215 (37.5)	193 (36.2)	105 (20.0)	115 (26.7)	
	Anaplastic	25 (14.1)	91 (15.9)	71 (13.3)	127 (24.1)	109 (25.3)	
AJCC clinical overall stage	I	45 (25.4)	56 (9.8)	56 (10.5)	0 (0.0)	0 (0.0)	<0.001
	II	120 (67.8)	280 (48.9)	145 (27.2)	350 (66.5)	0 (0.0)	
	III	12 (6.8)	233 (40.7)	295 (55.3)	137 (26.0)	367 (85.3)	
	IVA	0 (0.0)	4 (0.7)	37 (6.9)	39 (7.4)	63 (14.7)	
Regional nodes examined	<15	107 (60.5)	324 (56.5)	326 (61.2)	302 (57.4)	260 (60.5)	0.500
	≥15	70 (39.5)	249 (43.5)	207 (38.8)	224 (42.6)	170 (39.5)	
Margin status	Positive	24 (13.6)	53 (9.2)	33 (6.2)	13 (2.5)	4 (0.9)	<0.001
	Negative	153 (86.4)	520 (90.8)	500 (93.8)	513 (97.5)	426 (99.1)	
Lymphovascular invasion	Absent	63 (35.6)	248 (43.3)	255 (47.8)	219 (41.6)	160 (37.2)	<0.001
	Present	28 (15.8)	65 (11.3)	27 (5.1)	12 (2.3)	0 (0.0)	
	Unknown	86 (48.6)	260 (45.4)	251 (47.1)	295 (56.1)	270 (62.8)	
Length of stay	Median (i.q.r.)	9.0 (13.0)	10.0 (9.0)	9.0 (9.0)	9.0 (7.0)	9.0 (9.0)	0.500

Values are *n* (%) unless otherwise indicated. CCI, Charlson co-morbidity index; nCT, neoadjuvant chemotherapy; nCRT, neoadjuvant chemoradiotherapy; AJCC, American Joint Commission on Cancer; i.q.r., interquartile range.

#### Overall survival

Patients who had downstaged disease by three or more stages had significantly longer survival than those with downstaged disease by two stages or one stage and no change or upstaged disease (median of 78.8 *versus* 72.6 *versus* 43.6 *versus* 33.3 *versus* 25.8 months respectively, *P* < 0.001) (*Fig. 1b*). In adjusted analysis, patients with downstaged disease by three or more stages (HR 0.55, 95 per cent c.i. 0.43 to 0.71, *P* < 0.001), two stages (HR 0.58, 95 per cent c.i. 0.46 to 0.73, *P* < 0.001), or one stage (HR 0.69, 95 per cent c.i. 0.55 to 0.86, *P* = 0.001) had significantly longer survival than those with upstaged disease (*Table 2*). Other adverse independent prognostic factors were the presence of poor/anaplastic tumour grade, 15 or more lymph nodes examined, margin-negative resections, and absent lymphovascular invasion (*Table S5*). Sensitivity analyses were performed for both nCRT and nCT, which demonstrated consistent results in patients receiving nCRT, but not those receiving nCT (*Table S2*). Sensitivity analyses were performed by receipt of adjuvant therapy, which demonstrated consistent results (*Table S3*).

#### Sensitivity analysis by downstaging of T3/4 N+

**Fig. 3:**
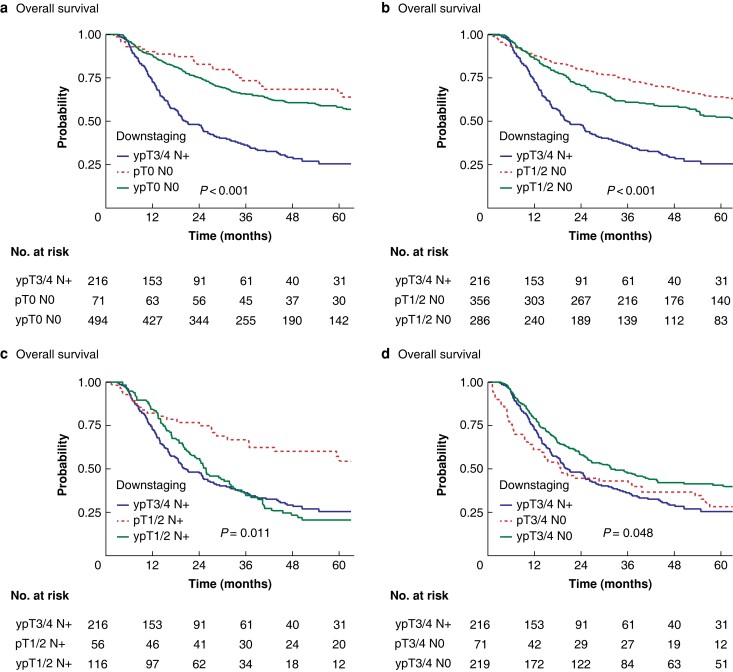
Comparison of patients with oesophageal squamous cell carcinoma receiving neoadjuvant therapy and oesophagectomy, downstaged from cT3/4 N+ **a** ypT0 N0/pT0 N0. **b** ypT1/2 N0/pT1/2 N0. **c** ypT1/2 N+/pT1/2 N+. **d** ypT3/4 N0/pT3/4 N0. Control groups are represented by cT3/4 N+ to ypT3/4 N+ (not downstaged) and stage-matched controls.

Sensitivity analyses of patients initially clinically staged as T3/4 N+ (cT3/4 N+) who received NAT and were downstaged to ypT0 N0 (*Fig. 3a*), ypT1/2 N0 (*Fig. 3b*), ypT1/2 N+ (*Fig. 3c*), or ypT3/4 N0 (*Fig. 3d*) were performed. In each survival graph, the two control curves represent stage-matched patients who were not administered nCT (pTNM) and patients who were not downstaged by chemotherapy (that is non-responders who were still ypT3/4 N+ after surgical resection). In all of these survival analyses, as seen in patients with OAC, a significant survival benefit was seen in NAT responders *versus* non-responders, whereas no difference was observed between responders and stage-matched neoadjuvant-naive controls (*Table 3*).

#### Sensitivity analysis by margin-negative resections

Sensitivity analyses in patients with margin-negative resections (2075 patients) demonstrated patients who had downstaged disease by three or more stages had significantly longer survival than those with downstaged disease by two stages or one stage and no change or upstaged disease (median of 78.8 *versus* 72.3 *versus* 47.7 *versus* 38.3 *versus* 28.0 months respectively, *P* < 0.001), consistent on adjusted analysis (*Table S6*). Similar results were seen in patients receiving nCRT and nCT (*Table S6*).

## Discussion

NAT followed by oesophagectomy remains the optimum curative treatment for patients with locally advanced oesophageal cancer^12,13^. However, this treatment is still associated with recurrence rates of more than 50 per cent^14–16^. In this setting, the prognostic value of the extent of downstaging has rarely been reported^3^. This national cohort study including almost 14 000 patients with OAC and OSCC demonstrated that downstaging by three or more stages is independently associated with improved long-term survival, both in patients receiving nCT and nCRT. Notably, even downstaging by one stage is associated with an improvement in survival by 19 and 17 per cent compared with upstaged disease for OAC and OSCC respectively. Further, there appeared to be similar survival in patients who received NAT compared with similarly staged neoadjuvant-naive patients, which was evident for both histological subtypes. These findings are similar to a single-centre study from the UK^3^. However, the previous study did not demonstrate this significance with OSCC and showed a survival advantage for patients who received NAT compared with unimodality surgery with an equivalent pathological stage, for advanced OAC and OSCC.

The findings from this study raise several important points for consideration. First, the increased downstaging in some patients might reflect a cohort of patients with tumours chemosensitive to specific chemotherapy regimens, and a further cohort of patients in which chemotherapy is ineffective. Second, recent studies have demonstrated a deleterious impact of neoadjuvant oncological therapy on fitness^17^. The impact of this decline of fitness on survival has not been established. However, whether the ability to predetermine the impact of NAT on disease stage based on specific biological factors of a tumour can help individualize oncological therapy is still a matter of debate. This may spare a subgroup of patients from the adverse physiological impact of neoadjuvant oncological therapy^18^. In these patients, as identified by this paper, NAT may lead to static or a poorer stage and no apparent improvement in survival. In future, approaches to deliver targeted therapy would include: analysis of tumour heterogeneity or clonality before NAT; non-invasive biomarkers of response, such as circulating tumour DNA; radiological assessment of response using PET-CT; improved systems to assess tumour regression^19^; and careful consideration of the role of surgery in patients whose disease regresses during preoperative chemotherapy, with an ultimate aim of organ preservation in a subgroup of patients^20^.

The Eighth Edition of the AJCC staging system includes both clinical and pathological stage grouping, but is imperfect. To maximize accuracy of staging, it relies on appropriate lymphadenectomy^21,22^. There is a difference between the lymphadenectomy required to help accurately stage the disease, and the lymphadenectomy required for optimal oncological control. A previous cohort study identified that accurate staging is dependent on tumour size, with short cancers (less than 2.5 cm) requiring a minimum of 20 nodes, whereas more extended cancers (greater than or equal to 2.5 cm) require approximately 60 lymph nodes^23^. However, the number of nodes required for oncological clearance is likely to be related to the depth of tumour invasion, with a stepwise increase in suggested nodes to be obtained with lymphadenectomy: T1 tumours requiring 10 nodes to be obtained, T2 tumours requiring 20 nodes to be obtained, and T3 tumours requiring more than 30 nodes to be obtained^24^.

This study has important limitations that need to be addressed. First, comparing pathological stage with post-neoadjuvant stage may misrepresent the impact of NAT. It is difficult to ascertain the inherent differences or inaccuracies in staging modalities and their use across different centres within the included cohort study and variation in staging approaches may reflect this^25^. For example, the use of endoscopic ultrasound and PET imaging are not routinely adopted in all of the centres. Second, the NCDB does not provide information on the chemotherapy regimens, such as FLOT and CROSS, and thus does not permit a deeper comparison of the impact of downstaging and data on Mandard regression gradings are not available. Again, the variation in use of these chemotherapy options is likely to reflect centre-level practices. Third, the NCDB does not capture all cancer cases (approximately 72 per cent) and there may be underlying selection bias within the included patients. Finally, this study lacks data on cancer-specific or recurrence-free survival to evaluate the impact of downstaging on these endpoints.

The extent of downstaging after NAT is an important prognostic factor in patients with both OAC and OSCC. Further, patients with an equivalent pathological stage who have received NAT have a more favourable prognosis, with important implications for counselling patients. However, the optimal NAT for both OAC and OSCC remains controversial. Identifying biomarkers associated with response to chemotherapy regimens is imperative in moving towards a more tailored treatment.

## Supplementary Material

zrad042_Supplementary_Data

## Data Availability

The data that support the findings of this study are available from the corresponding author upon reasonable request.
