# Supplementary material for: Prognostic implications of the extent of downstaging after neoadjuvant therapy for oesophageal adenocarcinoma and oesophageal squamous cell carcinoma
Source: BJS Open. 2023 Jun 21;7(3):zrad042. doi: 10.1093/bjsopen/zrad042 (PMC10282921; doi:10.1093/bjsopen/zrad042)
Supplement: zrad042_Supplementary_Data [file zrad042_supplementary_data.docx]

**Prognostic Implications on the Extent of Downstaging following Neoadjuvant Therapy for Oesophageal Adenocarcinoma and Squamous Cell Carcinoma**

Sivesh K Kamarajah,^1, 2^ Sheraz R Markar,^3, 4, 5^ Donald Low,^6^ Alexander W Phillips^7, 8^

1. Department of Upper Gastrointestinal Surgery, Queen Elizabeth Hospital Birmingham, University Hospitals Birmingham NHS Trust, Birmingham, United Kingdom
2. Institute of Cancer and Genomic Sciences, College of Medical and Dental Sciences, University of Birmingham, Birmingham
3. Department of Surgery & Cancer, Imperial College London, London, United Kingdom
4. Department of Molecular Medicine & Surgery, Karolinska Institutet, Stockholm, Sweden
5. Nuffield Department of Surgery, University of Oxford, United Kingdom
6. Department of Thoracic Surgery, Virginia Mason Medical Center, Seattle, WA
7. Northern Oesophagogastric Unit, Royal Victoria Infirmary, Newcastle University Trust Hospitals, Newcastle-Upon-Tyne, United Kingdom
8. School of Medical Education, Newcastle University, Newcastle upon Tyne, Tyne and Wear, United Kingdom

**Running title**: Downstaging in oesophageal cancers

**Corresponding Author**:

Mr Alexander Phillips

Northern Oesophagogastric Unit,

Royal Victoria Infirmary,

Newcastle University Trust Hospitals,

Newcastle-Upon-Tyne,

United Kingdom

**Supplementary Materials - Index**

| **Supplementary Figures and Tables** |  |
| --- | --- |
| Table S1 | *Page 3-4* |
| Table S2 | *Page 5* |
| Table S3 | *Page 6* |
| Table S4 | *Page 7* |
| Table S5 | *Page 8-9* |
| Table S6 | *Page 10* |
|  |  |

Table S1 Cox regression on overall survival in patients undergoing oesophagectomy for oesophageal adenocarcinoma by degree of downstaging

|  |  | HR (univariable) | HR (multivariable) |
| --- | --- | --- | --- |
| Downstaging | Upstaged | - | - |
|  | No change | 0.74 (0.67-0.81, p<0.001) | 0.76 (0.70-0.84, p<0.001) |
|  | Downstaged - 1 stage | 0.53 (0.48-0.58, p<0.001) | 0.57 (0.52-0.62, p<0.001) |
|  | Downstaged - 2 stage | 0.38 (0.34-0.42, p<0.001) | 0.43 (0.39-0.48, p<0.001) |
|  | Downstaged - >/=3 stage | 0.36 (0.33-0.40, p<0.001) | 0.40 (0.36-0.44, p<0.001) |
| Facility Type | Community | - | - |
|  | Integrated | 0.91 (0.84-0.98, p=0.012) | 0.99 (0.91-1.07, p=0.769) |
|  | Academic | 0.87 (0.83-0.92, p<0.001) | 0.90 (0.84-0.95, p<0.001) |
| Facility Location | Northeast | - | - |
|  | Midwest | 1.07 (1.00-1.14, p=0.041) | 1.02 (0.96-1.10, p=0.482) |
|  | South | 1.11 (1.04-1.19, p=0.002) | 1.03 (0.96-1.11, p=0.383) |
|  | West | 0.93 (0.86-1.01, p=0.104) | 0.91 (0.83-0.99, p=0.030) |
| Hospital Distance | <12.5 miles | - | - |
|  | 12.5-49.9 miles | 1.16 (1.10-1.23, p<0.001) | 1.10 (1.04-1.17, p=0.002) |
|  | >/=50 miles | 1.19 (1.12-1.27, p<0.001) | 1.12 (1.03-1.21, p=0.006) |
| Year of Diagnosis | 2004-2005 | - | - |
|  | 2006-2007 | 1.06 (0.93-1.20, p=0.386) | 1.04 (0.91-1.18, p=0.598) |
|  | 2008-2009 | 1.00 (0.88-1.13, p=0.963) | 1.00 (0.88-1.13, p=0.982) |
|  | 2010-2011 | 1.16 (1.04-1.30, p=0.009) | 1.19 (1.05-1.34, p=0.006) |
|  | 2012-2013 | 1.06 (0.94-1.18, p=0.344) | 1.10 (0.97-1.24, p=0.140) |
|  | 2014-2015 | 0.96 (0.85-1.08, p=0.491) | 1.02 (0.90-1.17, p=0.719) |
|  | 2016-2017 | 1.01 (0.90-1.13, p=0.838) | 1.09 (0.96-1.24, p=0.171) |
| Age at Diagnosis, years | 18-35 | - | - |
|  | 36-50 | 1.13 (0.82-1.56, p=0.453) | 1.14 (0.82-1.59, p=0.438) |
|  | 51-65 | 1.34 (0.98-1.83, p=0.064) | 1.39 (1.00-1.93, p=0.048) |
|  | 66-80 | 1.57 (1.15-2.14, p=0.005) | 1.58 (1.14-2.20, p=0.007) |
|  | 80+ | 2.52 (1.75-3.62, p<0.001) | 2.67 (1.82-3.91, p<0.001) |
| Sex | Male | - | - |
|  | Female | 0.79 (0.73-0.86, p<0.001) | 0.82 (0.75-0.89, p<0.001) |
| Race | White | - | - |
|  | Other | 0.89 (0.77-1.02, p=0.091) | 0.93 (0.81-1.07, p=0.327) |
| CDCC Score | 0 | - | - |
|  | 1-2 | 1.19 (1.12-1.25, p<0.001) | 1.14 (1.08-1.20, p<0.001) |
|  | 2+ | 1.28 (1.06-1.54, p=0.011) | 1.24 (1.02-1.50, p=0.028) |
| Insurance Status | Medicare | - | - |
|  | Medicaid | 1.03 (0.92-1.16, p=0.625) | 1.16 (1.02-1.31, p=0.026) |
|  | Private | 0.81 (0.77-0.85, p<0.001) | 0.92 (0.86-0.99, p=0.019) |
|  | Not Insured / Other | 0.92 (0.80-1.05, p=0.227) | 1.09 (0.94-1.25, p=0.262) |
| Education Level | >21% | - | - |
|  | 13%-20.9% | 1.53 (1.41-1.65, p<0.001) | 1.28 (1.18-1.40, p<0.001) |
|  | 7%-12.9% | 1.38 (1.28-1.48, p<0.001) | 1.28 (1.18-1.39, p<0.001) |
|  | <7% | 1.22 (1.13-1.32, p<0.001) | 1.26 (1.16-1.38, p<0.001) |
| Medical Income | </=$47,999 | - | - |
|  | $48,000-$62,999 | 0.97 (0.92-1.03, p=0.374) | 0.94 (0.88-1.00, p=0.061) |
|  | $63,000 + | 0.72 (0.68-0.76, p<0.001) | 0.78 (0.73-0.84, p<0.001) |
| Residence | Metro | - | - |
|  | Urban | 1.16 (1.09-1.24, p<0.001) | 1.02 (0.95-1.10, p=0.512) |
|  | Rural | 0.78 (0.70-0.86, p<0.001) | 0.76 (0.68-0.85, p<0.001) |
| Neoadjuvant Therapy | nCT | - | - |
|  | nCRT | 1.13 (1.02-1.25, p=0.019) | 1.32 (1.19-1.46, p<0.001) |
| Tumor Grade | Well | - | - |
|  | Moderate | 1.19 (1.04-1.36, p=0.012) | 1.19 (1.04-1.37, p=0.011) |
|  | Poor | 1.53 (1.34-1.75, p<0.001) | 1.48 (1.29-1.70, p<0.001) |
|  | Anaplastic | 1.13 (0.98-1.30, p=0.098) | 1.15 (1.00-1.33, p=0.057) |
| Regional Nodes Examined | <15 | - | - |
|  | >/=15 | 0.88 (0.83-0.92, p<0.001) | 0.86 (0.81-0.90, p<0.001) |
| Margin Status | Positive | - | - |
|  | Negative | 0.48 (0.44-0.53, p<0.001) | 0.63 (0.57-0.69, p<0.001) |
| Lymphovascular Invasion | Absent | - | - |
|  | Present | 1.73 (1.61-1.87, p<0.001) | 1.36 (1.25-1.47, p<0.001) |
|  | Unknown | 1.01 (0.96-1.07, p=0.650) | 1.04 (0.98-1.11, p=0.216) |
| Length of Stay | Mean (SD) | 1.01 (1.01-1.02, p<0.001) | 1.01 (1.01-1.02, p<0.001) |

Table S2 Adjusted multivariable Cox regression on overall survival in patients undergoing neoadjuvant therapy and oesophagectomy for oesophageal adenocarcinoma and squamous cell carcinoma by degree of downstaging stratified by type of neoadjuvant therapy (i.e. nCRT or nCT)

|  |  | **Patients, n** | **Overall survival, months** | **Hazard ratio (CI_95%_)** | **p-value** |
| --- | --- | --- | --- | --- | --- |
| **Adenocarcinoma** | | | | | |
| **nCRT** | Upstaged | 688 (6.5) | 21.1 (19.6 - 23.8) | REF |  |
|  | No change | 2848 (26.8) | 28.3 (26.8 - 29.9) | 0.76 (0.69-0.84) | <0.001 |
|  | Downstaged - 1 stage | 3166 (29.8) | 40.4 (37.0 - 43.0) | 0.57 (0.52-0.63) | <0.001 |
|  | Downstaged - 2 stage | 2011 (18.9) | 66.7 (59.7 - 73.3) | 0.43 (0.39-0.48) | <0.001 |
|  | Downstaged - >3 stage | 1912 (18.0) | 74.7 (64.0 - 89.2) | 0.40 (0.36-0.45) | <0.001 |
|  |  |  |  |  |  |
| **nCT** | Upstaged | 76 (10.4) | 24.7 (18.7 - 34.9) | REF |  |
|  | No change | 245 (33.6) | 29.4 (24.6 - 40.6) | 0.75 (0.54-1.02) | 0.1 |
|  | Downstaged - 1 stage | 217 (29.7) | 53.3 (47.2 - 72.4) | 0.54 (0.39-0.75) | <0.001 |
|  | Downstaged - 2 stage | 122 (16.7) | 99.9 (67.9 - NR) | 0.41 (0.27-0.62) | <0.001 |
|  | Downstaged - >3 stage | 70 (9.6) | 93.9 (45.9 - NR) | 0.42 (0.25-0.69) | 0.001 |
| **Squamous cell carcinoma** | | | | | |
| **nCRT** | Upstaged | 160 (7.5) | 25.8 (21.3 - 30.9) | REF |  |
|  | No change | 537 (25.3) | 33.3 (29.0 - 40.7) | 0.87 (0.70-1.09) | 0.2 |
|  | Downstaged - 1 stage | 498 (23.4) | 43.7 (37.9 - 60.2) | 0.70 (0.55-0.88) | 0.002 |
|  | Downstaged - 2 stage | 510 (24.0) | 72.3 (61.3 - 97.4) | 0.60 (0.47-0.76) | <0.001 |
|  | Downstaged - >3 stage | 421 (19.8) | 78.8 (69.4 - 114.1) | 0.57 (0.44-0.73) | <0.001 |
|  |  |  |  |  |  |
| **nCT** | Upstaged | 17 (15.0) | 22.4 (17.0 - NR) | REF |  |
|  | No change | 36 (31.9) | 37.9 (16.0 - NR) | 0.95 (0.32-2.79) | 1.0 |
|  | Downstaged - 1 stage | 35 (31.0) | 40.8 (23.8 - NR) | 0.90 (0.27-2.96) | 0.9 |
|  | Downstaged - 2 stage | 16 (14.2) | 134.8 (35.5 - NR) | 0.28 (0.06-1.32) | 0.1 |
|  | Downstaged - >3 stage | 9 (8.0) | 45.6 (25.7 - NR) | 0.22 (0.03-1.66) | 0.1 |

Table S3 Adjusted multivariable Cox regression on overall survival in patients undergoing neoadjuvant therapy and oesophagectomy for oesophageal adenocarcinoma and squamous cell carcinoma by degree of downstaging stratified by receipt of adjuvant therapy

|  |  | **Patients, n** | **Overall survival, months** | **Hazard ratio (CI_95%_)** | **p-value** |
| --- | --- | --- | --- | --- | --- |
| **Adenocarcinoma** | | | | | |
| **No Adjuvant therapy** | Upstaged | 638 (6.2) | 21 (19.2 - 23.5) | REF |  |
|  | No change | 2650 (25.9) | 27.5 (26.3 - 29.4) | 0.78 (0.71 - 0.87) | <0.001 |
|  | Downstaged - 1 stage | 3064 (29.9) | 41.3 (38.1 - 44.4) | 0.57 (0.52 - 0.64) | <0.001 |
|  | Downstaged - 2 stage | 2001 (19.6) | 66.7 (62.3 - 74.6) | 0.44 (0.39 - 0.49) | <0.001 |
|  | Downstaged - >3 stage | 1881 (18.4) | 74.6 (63.5 - 88.3) | 0.41 (0.36 - 0.46) | <0.001 |
|  |  |  |  |  |  |
| **Adjuvant therapy** | Upstaged | 126 (11.2) | 25.8 (20.2 - 33.8) | REF |  |
|  | No change | 443 (39.5) | 32.5 (29.4 - 40.4) | 0.68 (0.53 - 0.86) | 0.002 |
|  | Downstaged - 1 stage | 319 (28.5) | 41.3 (34.5 - 52) | 0.57 (0.44 - 0.74) | <0.001 |
|  | Downstaged - 2 stage | 132 (11.8) | 103.2 (58.7 - NR) | 0.42 (0.30 - 0.59) | <0.001 |
|  | Downstaged - >3 stage | 101 (9.0) | 138.6 (54 - NR) | 0.31 (0.21 - 0.46) | <0.001 |
| **Squamous cell carcinoma** | | | | | |
| **No Adjuvant therapy** | Upstaged | 156 (7.4) | 24.0 (19.8 - 30.4) | REF |  |
|  | No change | 521 (24.8) | 32.1 (27.9 - 39.2) | 0.85 (0.68-1.07) | 0.2 |
|  | Downstaged - 1 stage | 506 (24.1) | 43.6 (38.9 - 60.2) | 0.66 (0.52-0.83) | <0.001 |
|  | Downstaged - 2 stage | 502 (23.9) | 72.3 (60.2 - 97.4) | 0.56 (0.44-0.72) | <0.001 |
|  | Downstaged - >3 stage | 415 (19.8) | 78.8 (69.4 - 114.1) | 0.53 (0.41-0.69) | <0.001 |
|  |  |  |  |  |  |
| **Adjuvant therapy** | Upstaged | 21 (15.1) | 34.8 (23.7 - NR) | REF |  |
|  | No change | 52 (37.4) | 49.5 (31.8 - NR) | 0.87 (0.35-2.15) | 0.8 |
|  | Downstaged - 1 stage | 27 (19.4) | 26.7 (20.2 - NR) | 1.63 (0.58-4.59) | 0.4 |
|  | Downstaged - 2 stage | 24 (17.3) | 107.8 (64.4 - NR) | 0.62 (0.20-1.86) | 0.4 |
|  | Downstaged - >3 stage | 15 (10.8) | 34.5 (22.6 - NR) | 0.83 (0.24-2.89) | 0.8 |

Table S4 Adjusted multivariable Cox regression on overall survival in patients undergoing neoadjuvant therapy and oesophagectomy for oesophageal adenocarcinoma by degree of downstaging stratified by type of neoadjuvant therapy (i.e. nCRT or nCT)

|  |  | **Patients, n** | **Overall survival, months** | **Hazard ratio (CI_95%_)** | **p-value** |
| --- | --- | --- | --- | --- | --- |
| **Overall** | Upstaged | 638 (5.9) | 23.4 (21.0 - 25.6) | REF |  |
|  | No change | 2822 (26.3) | 29.6 (28.0 - 31.1) | 0.78 (0.71-0.86) | <0.001 |
|  | Downstaged - 1 stage | 3218 (30.0) | 42.5 (40.2 - 46.7) | 0.57 (0.52-0.64) | <0.001 |
|  | Downstaged - 2 stage | 2084 (19.4) | 67.9 (63.1 - 76.9) | 0.44 (0.39-0.49) | <0.001 |
|  | Downstaged - >3 stage | 1969 (18.3) | 77.0 (65.3 - 89.5) | 0.40 (0.36-0.45) | <0.001 |
|  |  |  |  |  |  |
| **nCRT** | Upstaged | 578 (5.7) | 23.0 (20.9 - 25.6) | REF |  |
|  | No change | 2602 (25.9) | 29.6 (27.9 - 31.1) | 0.78 (0.70-0.87) | <0.001 |
|  | Downstaged - 1 stage | 3012 (29.9) | 41.5 (38.4 - 45.0) | 0.58 (0.52-0.64) | <0.001 |
|  | Downstaged - 2 stage | 1966 (19.5) | 66.7 (61.4 - 74.6) | 0.44 (0.39-0.50) | <0.001 |
|  | Downstaged - >3 stage | 1900 (18.9) | 74.7 (64.4 - 89.2) | 0.40 (0.36-0.46) | <0.001 |
|  |  |  |  |  |  |
| **nCT** | Upstaged | 60 (8.9) | 25.8 (18.7 - 38.5) | REF |  |
|  | No change | 220 (32.7) | 31.8 (24.6 - 42.4) | 0.78 (0.54-1.10) | 0.2 |
|  | Downstaged - 1 stage | 206 (30.6) | 55.5 (47.3 - 74.3) | 0.53 (0.37-0.76) | 0.001 |
|  | Downstaged - 2 stage | 118 (17.5) | NR (67.9 - NR) | 0.39 (0.25-0.61) | <0.001 |
|  | Downstaged - >3 stage | 69 (10.3) | 93.9 (77.2 - NR) | 0.41 (0.24-0.70) | 0.001 |

Table S5 Cox regression on overall survival in patients undergoing neoadjuvant therapy and oesophagectomy for oesophageal squamous cell carcinoma by degree of downstaging

|  |  | HR (univariable) | HR (multivariable) |
| --- | --- | --- | --- |
| Downstaging | Upstaged | - | - |
|  | No change | 0.84 (0.69-1.03, p=0.094) | 0.86 (0.69-1.06, p=0.148) |
|  | Downstaged - 1 stage | 0.67 (0.54-0.82, p<0.001) | 0.69 (0.55-0.86, p=0.001) |
|  | Downstaged - 2 stage | 0.52 (0.42-0.65, p<0.001) | 0.58 (0.46-0.73, p<0.001) |
|  | Downstaged - >/=3 stage | 0.49 (0.39-0.62, p<0.001) | 0.55 (0.43-0.71, p<0.001) |
| Facility Type | Community | - | - |
|  | Integrated | 0.96 (0.80-1.15, p=0.628) | 1.09 (0.90-1.32, p=0.385) |
|  | Academic | 0.88 (0.77-1.00, p=0.045) | 0.86 (0.75-0.99, p=0.031) |
| Facility Location | Northeast | - | - |
|  | Midwest | 1.08 (0.92-1.26, p=0.350) | 0.95 (0.80-1.12, p=0.510) |
|  | South | 1.19 (1.03-1.38, p=0.021) | 1.10 (0.94-1.29, p=0.235) |
|  | West | 0.94 (0.78-1.15, p=0.575) | 0.87 (0.71-1.08, p=0.208) |
| Hospital Distance | <12.5 miles | - | - |
|  | 12.5-49.9 miles | 1.16 (1.02-1.32, p=0.022) | 1.13 (0.98-1.29, p=0.092) |
|  | >/=50 miles | 1.22 (1.05-1.41, p=0.010) | 1.21 (1.01-1.46, p=0.043) |
| Year of Diagnosis | 2004-2005 | - | - |
|  | 2006-2007 | 0.98 (0.75-1.27, p=0.876) | 0.99 (0.76-1.29, p=0.937) |
|  | 2008-2009 | 0.98 (0.75-1.27, p=0.857) | 1.11 (0.85-1.46, p=0.429) |
|  | 2010-2011 | 1.05 (0.83-1.32, p=0.685) | 1.14 (0.89-1.47, p=0.306) |
|  | 2012-2013 | 0.95 (0.75-1.19, p=0.632) | 1.08 (0.83-1.39, p=0.568) |
|  | 2014-2015 | 0.95 (0.73-1.23, p=0.701) | 1.09 (0.82-1.46, p=0.557) |
|  | 2016-2017 | 0.85 (0.66-1.08, p=0.178) | 0.99 (0.75-1.31, p=0.963) |
| Age at Diagnosis, years | 18-35 | - | - |
|  | 36-50 | 2.07 (0.84-5.07, p=0.113) | 1.98 (0.78-5.02, p=0.148) |
|  | 51-65 | 2.31 (0.96-5.58, p=0.062) | 2.15 (0.86-5.37, p=0.101) |
|  | 66-80 | 2.49 (1.03-6.01, p=0.043) | 2.12 (0.84-5.34, p=0.112) |
|  | 80+ | 2.89 (1.04-8.02, p=0.042) | 2.48 (0.85-7.18, p=0.095) |
| Sex | Male | - | - |
|  | Female | 0.84 (0.75-0.95, p=0.004) | 0.84 (0.75-0.95, p=0.006) |
| Race | White | - | - |
|  | Other | 0.97 (0.85-1.11, p=0.647) | 0.94 (0.81-1.09, p=0.406) |
| CDCC Score | 0 | - | - |
|  | 1-2 | 1.24 (1.08-1.42, p=0.002) | 1.18 (1.03-1.36, p=0.019) |
|  | 2+ | 0.95 (0.58-1.55, p=0.833) | 0.95 (0.57-1.56, p=0.828) |
| Insurance Status | Medicare | - | - |
|  | Medicaid | 0.89 (0.72-1.09, p=0.252) | 0.95 (0.75-1.21, p=0.693) |
|  | Private | 0.81 (0.71-0.91, p=0.001) | 0.83 (0.71-0.98, p=0.026) |
|  | Not Insured / Other | 1.07 (0.81-1.42, p=0.634) | 1.13 (0.84-1.54, p=0.419) |
| Education Level | >21% | - | - |
|  | 13%-20.9% | 1.51 (1.28-1.79, p<0.001) | 1.42 (1.19-1.69, p<0.001) |
|  | 7%-12.9% | 1.32 (1.13-1.55, p=0.001) | 1.30 (1.09-1.56, p=0.003) |
|  | <7% | 1.29 (1.09-1.53, p=0.003) | 1.37 (1.12-1.67, p=0.002) |
| Medical Income | </=$47,999 | - | - |
|  | $48,000-$62,999 | 1.07 (0.93-1.23, p=0.353) | 1.00 (0.85-1.18, p=0.999) |
|  | $63,000 + | 0.83 (0.73-0.94, p=0.005) | 0.88 (0.74-1.04, p=0.129) |
| Residence | Metro | - | - |
|  | Urban | 1.20 (1.02-1.40, p=0.029) | 1.07 (0.89-1.29, p=0.446) |
|  | Rural | 0.92 (0.72-1.18, p=0.524) | 0.90 (0.69-1.17, p=0.425) |
| Neoadjuvant Therapy | nCT | - | - |
|  | nCRT | 0.95 (0.74-1.22, p=0.672) | 1.07 (0.83-1.39, p=0.590) |
| Tumor Grade | Well | - | - |
|  | Moderate | 1.43 (1.10-1.85, p=0.007) | 1.32 (1.01-1.72, p=0.042) |
|  | Poor | 1.66 (1.27-2.16, p<0.001) | 1.50 (1.14-1.97, p=0.004) |
|  | Anaplastic | 1.40 (1.06-1.85, p=0.017) | 1.36 (1.02-1.80, p=0.036) |
| Regional Nodes Examined | <15 | - | - |
|  | >/=15 | 0.81 (0.72-0.91, p<0.001) | 0.83 (0.73-0.94, p=0.003) |
| Margin Status | Positive | - | - |
|  | Negative | 0.54 (0.43-0.67, p<0.001) | 0.62 (0.50-0.78, p<0.001) |
| Lymphovascular Invasion | Absent | - | - |
|  | Present | 1.93 (1.54-2.42, p<0.001) | 1.72 (1.36-2.17, p<0.001) |
|  | Unknown | 0.96 (0.85-1.08, p=0.498) | 1.00 (0.86-1.16, p=0.983) |
| Length of Stay | Mean (SD) | 1.01 (1.01-1.01, p<0.001) | 1.01 (1.01-1.01, p<0.001) |

Table S6 Adjusted multivariable Cox regression on overall survival in patients undergoing neoadjuvant therapy and oesophagectomy for oesophageal adenocarcinoma by degree of downstaging stratified by type of neoadjuvant therapy (i.e. nCRT or nCT)

|  |  | **Patients, n** | **Overall survival, months** | **Hazard ratio (CI_95%_)** | **p-value** |
| --- | --- | --- | --- | --- | --- |
| **Overall** | Upstaged | 146 (7.0) | 28.0 (23.7 - 35.3) | REF |  |
|  | No change | 511 (24.6) | 38.3 (32.2 - 45.5) | 0.82 (0.65-1.04) | 0.1 |
|  | Downstaged - 1 stage | 489 (23.6) | 47.7 (39.5 - 67.2) | 0.67 (0.53-0.85) | 0.001 |
|  | Downstaged - 2 stage | 509 (24.5) | 72.3 (61.3 - 88.9) | 0.60 (0.47-0.77) | <0.001 |
|  | Downstaged - >3 stage | 420 (20.2) | 78.8 (68.6 - 114.1) | 0.55 (0.43-0.71) | <0.001 |
|  |  |  |  |  |  |
| **nCRT** | Upstaged | 130 (6.6) | 28.0 (24.0 - 35.3) | REF |  |
|  | No change | 482 (24.4) | 38.5 (31.8 - 44.9) | 0.83 (0.65-1.06) | 0.1 |
|  | Downstaged - 1 stage | 457 (23.1) | 52.0 (39.5 - 67.5) | 0.67 (0.52-0.87) | 0.002 |
|  | Downstaged - 2 stage | 495 (25.1) | 72.0 (61.3 - 88.9) | 0.61 (0.48-0.79) | <0.001 |
|  | Downstaged - >3 stage | 411 (20.8) | 78.8 (68.6 - 114.1) | 0.56 (0.43-0.74) | <0.001 |
|  |  |  |  |  |  |
| **nCT** | Upstaged | 16 (16.0) | 28.6 (17.0 - NR) | REF |  |
|  | No change | 29 (29.0) | 38.3 (23.9 - NR) | 0.44 (0.13-1.47) | 0.2 |
|  | Downstaged - 1 stage | 32 (32.0) | 41.1 (23.8 - NR) | 0.60 (0.15-2.35) | 0.5 |
|  | Downstaged - 2 stage | 14 (14.0) | 79.9 (28.4 - NR) | 0.20 (0.03-1.19) | 0.1 |
|  | Downstaged - >3 stage | 9 (9.0) | 45.6 (25.7 - NR) | 0.13 (0.01-1.42) | 0.1 |
